# Three-dimensional analysis of the thoracic aorta microscopic deformation during intraluminal pressurization

**DOI:** 10.1007/s10237-019-01201-w

**Published:** 2019-07-11

**Authors:** Shukei Sugita, Masaya Kato, Fukui Wataru, Masanori Nakamura

**Affiliations:** Biomechanics Laboratory, Department of Electrical and Mechanical Engineering, Graduate School of Engineering, Nagoya Institute of Technology, Gokiso-cho, Showa-ku, Nagoya, 466-8555 Japan

**Keywords:** Microscale strain, Mouse thoracic aorta, Two-photon microscopy, Photo-bleaching, 3D strain tensor

## Abstract

The aorta is composed of various constituents with different mechanical properties. This heterogeneous structure implies non-uniform deformation in the aorta, which could affect local cell functions. The present study investigates 3D strains of the aorta at a cell scale induced by intraluminal pressurization. After resected mouse, thoracic aortas were stretched to their in vivo length, and the aortas were pressurized at 15, 40, 80, 120, and 160 mmHg. Images of autofluorescent light of elastin were captured under a two-photon microscope. From the movement of markers in elastic laminas (ELs) created by photo-bleaching, 3D strains (*ε*_*θθ*_, *ε*_*zz*_, *ε*_*rr*_, *ε*_*rθ*_, *ε*_*rz*_, *ε*_*θz*_) between two neighboring ELs in the circumferential (*θ*), longitudinal (*z*), and radial (*r*) directions with reference to the dimensions at 15 mmHg were calculated. The results demonstrated that the average of shear strain *ε*_*rθ*_ was almost 0 in a physiological pressure range (from 80 to 120 mmHg) with an absolute value |*ε*_*rθ*_| changing approximately by 5%. This indicates that ELs experience radial–circumferential shear at the cell scale, but not at the whole tissue scale. The normal strains in the circumferential *ε*_*θθ*_ and longitudinal direction *ε*_*zz*_ were positive but that in the radial direction *ε*_*rr*_ was almost 0, which demonstrates that aortic tissue is not an incompressible material. The first principal direction in the radial–circumferential plane was 29° ± 13° from the circumferential direction. We show that the aorta is not simply stretched in the circumferential direction during pressurization and that cells in the aorta undergo complex deformations by nature.

**Electronic supplementary material:**

The online version of this article (10.1007/s10237-019-01201-w) contains supplementary material, which is available to authorized users.

## Introduction

The aortic media is composed of two types of alternating layers in the radial direction: smooth muscle-rich layers (SMLs) and elastic laminas (ELs). SMLs are mainly composed of smooth muscle cells (SMCs) and collagen fibers, while ELs are mainly composed of elastin and collagen fibers. The elastic moduli of these constituents are remarkably different: The elastic modulus of collagen fibers is approximately 1 GPa (Fung 1981), approximately 0.6 MPa for elastin (Fung 1981), and 1–100 kPa for SMCs (Nagayama and Matsumoto 2004). Thus, the aorta is mechanically heterogeneous in the radial direction. The circumferential and longitudinal directions display heterogeneity as well. Longitudinally, the volume fraction of collagen in the descending thoracic aorta increases with the distance from the heart (Roveri et al. 1980). Circumferentially, alignment consistency and/or volume fraction of collagen fibers in the dorsal side is different from that in the ventral side (Sugita and Matsumoto 2013).

In nature, cells in the aorta perform various functions in response to the mechanical environment. Hydrostatic pressure works on the intraluminal surface and influences Ca^2+^ response (Ohashi et al. 2003), endothelial cell morphology, and expression of VE-cadherin of endothelial cells (Ohashi et al. 2003, 2007). A change in the hydrostatic pressure during a cardiac cycle causes a cyclic circumferential stretch of the aorta. During this cyclic stretch, both endothelial cells and SMCs in the aorta undergo profound changes in cell phenotype and function such as proliferation, apoptosis, and gene expression (Qiu et al. 2014). Since an elevated cyclic stretch increases expression and activity levels of proteolytic enzymes in SMCs in vitro (Balachandran et al. 2009), the cyclic stretch might induce aorta remodeling. The aorta is stretched in vivo in the longitudinal direction (Learoyd and Taylor 1966), and the effect of this longitudinal stretch on cells remains unclear.

A heterogeneous structure implies non-uniform deformations in the aorta. For instance, a circumferential stretch can be higher in the ventral than in the dorsal side (Draney et al. 2002; Sugita et al. 2003), because collagen fibers, which have the highest elastic modulus in the aorta (Fung 1981), are richer in the dorsal side (Sugita and Matsumoto 2013). We previously found that, in a low-pressure range, collagen fibers in SML were straightened, whereas those in EL stayed wavy (Sugita and Matsumoto 2017). Based on this finding, we concluded that SMLs are more stretched than ELs. Since the mechanical environment affects cellular functions, the heterogeneity of tissue mechanical properties may affect the local deformation of tissues and cell functions.

In the present study, we aimed to measure the 3D strain in the aorta at a cellular scale during intraluminal pressurization to evaluate the actual deformation of tissues around SMCs. Strain markers were created in ELs of an aorta tubular segment, and displacements of the markers were measured. From the displacement, 3D strain tensors were calculated.

## Materials and methods

### Sample preparation

Four Slc:ddY mice (8-week-old, 34–36 g, Chubu Kagaku Shizai, Nagoya, Japan) were used as a test model. All animal experiments were performed with the agreement of the institutional review board for animal care at Nagoya Institute of Technology and in accordance with the Guide for Animal Experimentation, Nagoya Institute of Technology. The mice were euthanized in a CO_2_ chamber. After exposing the thoracic aorta, gentian violet dots at 3-mm interval were made on the ventral side of the surface of the aorta as in vivo length markers, and intercostal arteries were cauterized. The aortas were then resected and kept in Krebs–Henseleit (KH) buffer (CaCl_2_2H_2_O, 2.3 mM; NaCl, 115.3 mM; KCl, 4.6 mM; MgSO_4_7H_2_O, 1.1 mM; NaHCO_3_, 22.1 mM; KH_2_PO_4_, 1.1 mM; glucose, 7.8 mM) at 4 °C until experiments.

### Pressure–diameter test

The pressure–diameter test was performed based on the previous study (Sugita and Matsumoto 2017). Both proximal and distal sides of the tubular aorta were tied to a 22-G hypodermic needle with suture threads so that its ventral side was top through the specimen as reference to the gentian violet markers. The aorta was stretched to its in vivo length with reference to the gentian violet markers. The specimen was pressurized with an electro-pneumatic regulator (640BA20B, Asahi Enterprise, Tokyo, Japan). The regulator was controlled using a software (NI LabVIEW 2010, National Instruments, Austin, TX, USA) installed on a personal computer (FMV BIBLO, Fujitsu, Tokyo, Japan; PC) through a digital–analog (D/A) converter (NI USB-6363, National Instruments). The intraluminal pressure of the specimen was measured with a pressure transducer (DX-300, Nihon Kohden, Tokyo, Japan), a strain amplifier (DPM-911B, Kyowa Electronic Instruments, Chofu, Japan), an analog–digital (A/D) converter (NI USB-6363, National Instruments), and the PC.

### Two-photon and second-harmonic generation light microscopy

A two-photon microscope (FV1200MPE, Olympus, Tokyo, Japan) was used for elastin and collagen imaging. In brief, an 800-nm Ti/sapphire laser was applied to specimens. Autofluorescent light of elastin and second-harmonic generation (SHG) light of collagen fibers were observed in a backward direction through 495–540 nm and 400 ± 5 nm band-pass filters, respectively. Images were captured through a 60 × objective lens (LUMPLFLN60XW, Olympus). Please refer to Sugita and Matsumoto for additional details (Sugita and Matsumoto 2017).

### Experimental protocol

Preconditioning was performed five times in a pressure range of 0–160 mmHg, at a rate of 2 mmHg/s. Elastin and collagen fibers were imaged from the intimal to the adventitial side with an interval of 2 µm. The position of the intimal side was confirmed from fenestrations at the internal elastic lamina (Campbell and Roach 1981). The dwell time was 4 µs/pixel, and the image size was 512 × 512 pixel. Spatial resolution was 0.414 µm/pixel. Laser power was regulated to 25% when the 5× objective lens was used and 3–30% when the 60× objective lens was used.

**Fig. 1:**
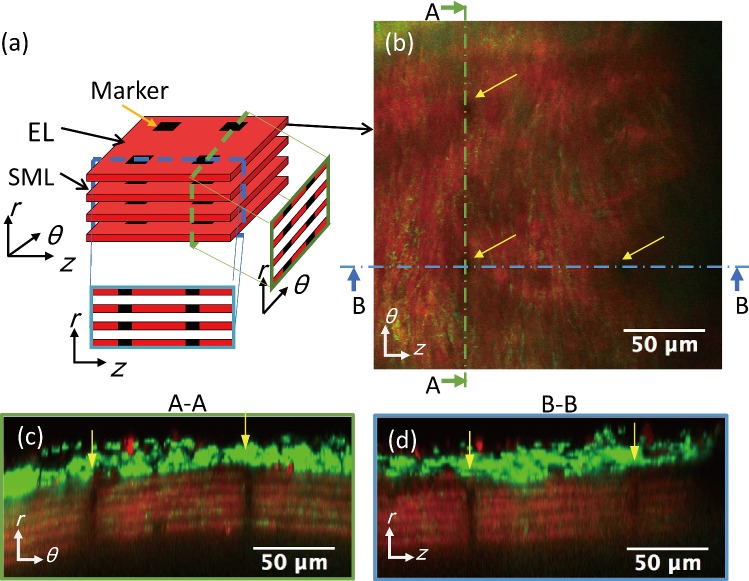
Markers created in ELs. **a** A schematic illustration of markers in ELs. **b**–**d** A typical two-photon image of elastin (red) and collagen (green) fibers captured with a 60× objective lens at 15 mmHg. **b** Image in the longitudinal–circumferential plane. **c**, **d** Resliced images of **c** A–A and **d** B–B lines in (**b**). Yellow arrows show the photo-bleached markers. Image contrast was adjusted for clear visibility

To observe the deformation of tissues, we adopted the technique of Jayyosi et al. (Jayyosi et al. 2014). Four strain markers were created by photo-bleaching at the vertices of a 100 × 100 µm^2^ square in each EL as shown in Fig. 1. While a pressure of 15 mmHg was applied to the intraluminal side of a specimen, a laser focus was set at the internal elastic lamina. The image was optically zoomed 50× under the 60× objective lens. Autofluorescent light of elastin was photo-bleached by applying the laser at half the power used for the observation. Photo-bleaching was stopped when the intensity was halved from the initial intensity or after 10-min exposure. This was repeated to create markers in all ELs.

Wrinkles were present in ELs at 0 mmHg of intraluminal pressure. Thus, we started to take images from a pressure of 15 mmHg at which the wrinkles had vanished. Elastin and collagen fibers in the circumferential–longitudinal plane were imaged at the pressure of 15, 40, 80, 120, and 160 mmHg. Before imaging, the specimen was held 1–2 min to finalize its viscoelastic deformation.

### Image analysis

Images were analyzed using the image analysis software ImageJ (v. 1.51i, National Institutes of Health, Bethesda, MD, USA). Captured image stacks were preprocessed as shown in Fig. S1.1. To evaluate tissue deformation, nodes to be tracked in bleached markers were defined as explained in Supplemental Material S2.

### Strain analysis

Strains (*ε*_*θθ*_, *ε*_*rr*_, *ε*_*rθ*_), (*ε*_*zz*_, *ε*_*rz*_), and *ε*_*θz*_ were obtained in radial–circumferential, radial–longitudinal, and circumferential–longitudinal planes, respectively, using isoparametric mapping with a shape function of the first order (see Supplemental Material S3 for details). Incremental strains Δ*ε*_*mn*_(*P*_*i*−1_, *P*_*i*_) with *m* and *n* = *θ*, *r*, and *z* were calculated from displacements of the nodes between the consecutive pressures (*P*_0_ = 15, *P*_1_ = 40, *P*_2_ = 80, *P*_3_ = 120, and *P*_4_ = 160 mmHg). Cumulative strain *ε*_*mn*_(*P*_*i*_) from 15 mmHg was calculated as:

1 $$\varepsilon_{mn} \left( {P_{i} } \right) = \left\{ {1 + \varepsilon_{mn} \left( {P_{i - 1} } \right)} \right\}\left\{ {1 + \Delta \varepsilon_{mn} \left( {P_{i - 1} ,\,P_{i} } \right)} \right\} - 1$$

where *ε*_*mn*_(*P*_*i*−1_) is the cumulative strain at the pressure in a previous step.

The first and second principal strains *ε*_1_, *ε*_2_, and the angle of the first principal direction from the circumferential direction *α*_1_ in the radial–circumferential plane were calculated from *ε*_*θθ*_, *ε*_*rr*_, and *ε*_*rθ*_ as

2 $$\left\{ {\begin{array}{*{20}c} {\varepsilon_{1} } \\ {\varepsilon_{2} } \\ {\alpha_{1} } \\ \end{array} } \right\} = \left\{ {\begin{array}{*{20}c} {\frac{{\varepsilon_{rr} + \varepsilon_{\theta \theta } }}{2} + \sqrt {\left( {\frac{{\varepsilon_{rr} - \varepsilon_{\theta \theta } }}{2}} \right)^{2} + \varepsilon_{r\theta }^{2} } } \\ {\frac{{\varepsilon_{rr} + \varepsilon_{\theta \theta } }}{2} - \sqrt {\left( {\frac{{\varepsilon_{rr} - \varepsilon_{\theta \theta } }}{2}} \right)^{2} + \varepsilon_{r\theta }^{2} } } \\ {\frac{1}{2}\text{Arctan} \frac{{2\varepsilon_{r\theta } }}{{\varepsilon_{rr} - \varepsilon_{\theta \theta } }}} \\ \end{array} } \right\}$$

Incremental first and second principal strains Δ*ε*_1_, Δ*ε*_2_, and the angle of the first principal direction *β*_1_ at *P*_*i*_ defined in reference to configuration at *P*_*i*−1_ were obtained in a similar fashion.

3 $$\left\{ {\begin{array}{*{20}c} {\Delta \varepsilon_{1} } \\ {\Delta \varepsilon_{2} } \\ {\beta_{1} } \\ \end{array} } \right\} = \left\{ {\begin{array}{*{20}c} {\frac{{\Delta \varepsilon_{rr} + \Delta \varepsilon_{\theta \theta } }}{2} + \sqrt {\left( {\frac{{\Delta \varepsilon_{rr} - \Delta \varepsilon_{\theta \theta } }}{2}} \right)^{2} + \Delta \varepsilon_{r\theta }^{2} } } \\ {\frac{{\Delta \varepsilon_{rr} + \Delta \varepsilon_{\theta \theta } }}{2} - \sqrt {\left( {\frac{{\Delta \varepsilon_{rr} - \Delta \varepsilon_{\theta \theta } }}{2}} \right)^{2} + \Delta \varepsilon_{r\theta }^{2} } } \\ {\frac{1}{2}\text{Arctan} \frac{{2\Delta \varepsilon_{r\theta } }}{{\Delta \varepsilon_{rr} - \Delta \varepsilon_{\theta \theta } }}} \\ \end{array} } \right\}$$

### Statistical method

Strains biased from 0 were evaluated with *t* test. Student’s unpaired *t* test was used for comparison between strains in SMLs and ELs. A significant level of 0.05 was used. The numbers *N* and *n* represent the number of mice and the total number of the available data, respectively. The data were averaged for *n* and shown as mean ± standard deviation (SD).

## Results

### Movement of strain markers

**Fig. 2:**
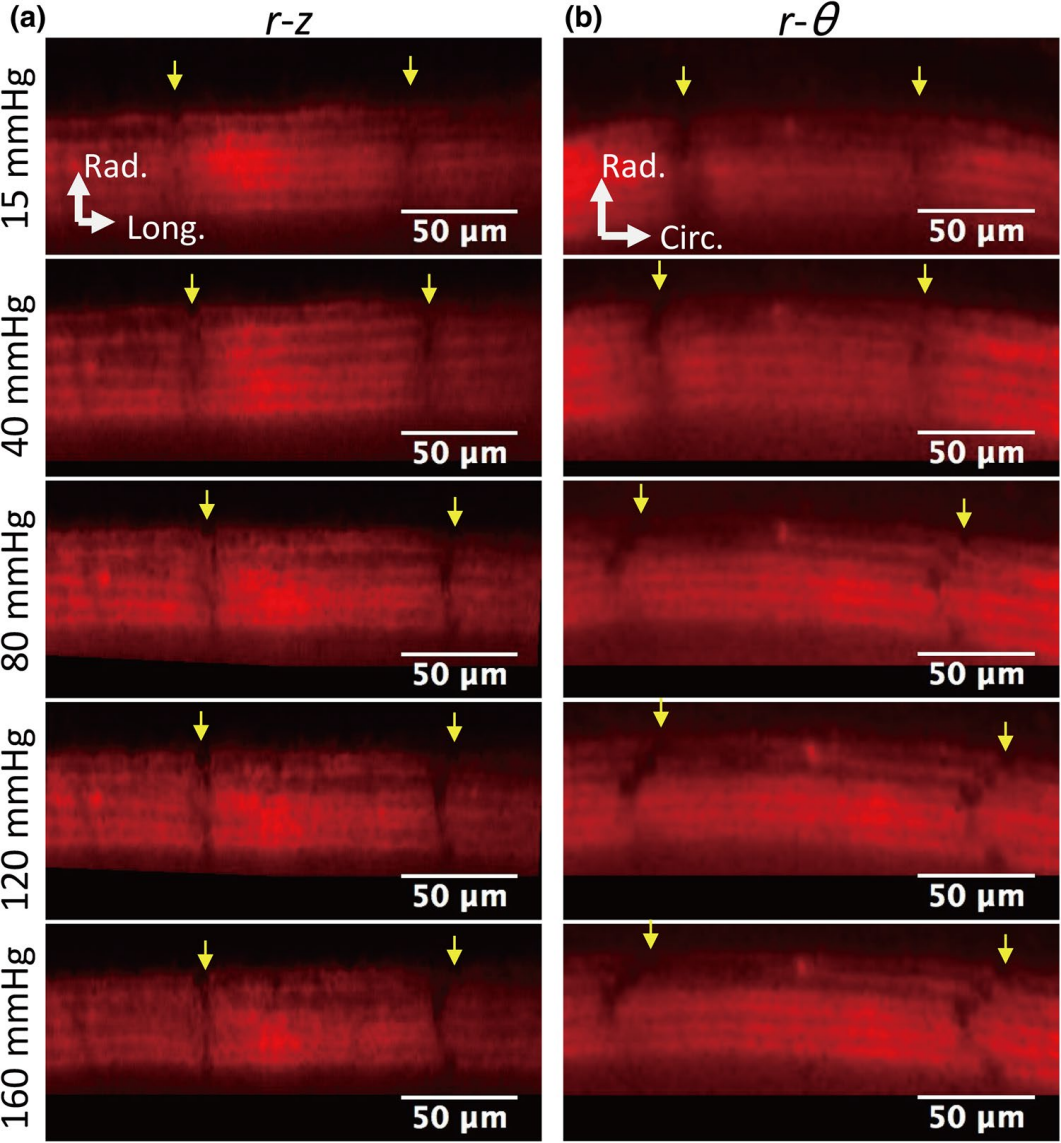
Deformation of ELs during pressurization. Typical images of **a** the radial–longitudinal and **b** radial–circumferential plane. Yellow arrows indicate the markers at outermost ELs. Image contrast was adjusted for clear visibility

Figure 2 and Movie 1 show typical images of elastin fibers in the radial–longitudinal (Fig. 2a) and radial–circumferential planes (Fig. 2b) of the aorta. Most of the strain markers were identifiable, and displacements of ELs during pressurization were observed. In the radial–longitudinal plane, the strain markers stayed in almost the same position after pressurization. In the radial–circumferential plane, markers in the same EL came to be circumferentially apart with pressurization. The strain markers showed different movements in the circumferential direction from EL to EL.

### Normal strains

**Fig. 3:**
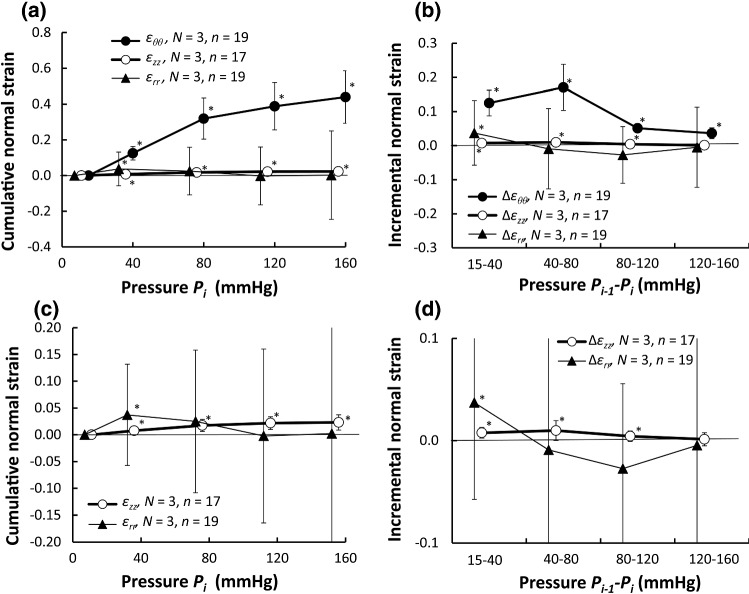
Changes in normal strains during pressurization. Cumulative and incremental normal strains are presented in **a**, **b**, respectively. Their magnified graph in vertical axes is shown in **c**, **d**, respectively. *ε*_*θθ*_: circumferential normal strain, *ε*_*zz*_: longitudinal normal strain, *ε*_*rr*_: radial normal strain. Incremental strains are expressed with Δ. *, *P* < 0.05 vs strain 0. Data are shown as mean ± SD

Figure 3 shows cumulative and incremental normal strains during pressurization. The cumulative normal strain in the circumferential direction *ε*_*θθ*_ increased with the pressurization (Fig. 3a), whereas its incremental normal strain Δ*ε*_*θθ*_ gradually decreased (Fig. 3b), showing a nonlinear mechanical property of the aorta. All cumulative and incremental normal strains in the circumferential direction were significantly larger than strain 0. The strain *ε*_*θθ*_ was not so different between different radial positions (Fig. S5.1a).

The cumulative normal strain in the longitudinal direction *ε*_*zz*_ was almost zero (Fig. 3a, c). This is reasonable because the longitudinal direction of the specimen was fixed in this study. A statistical comparison, however, showed a significant difference compared to zero strain, suggesting an increase with pressurization, although the degree of the increase was far smaller than the normal strain in the circumferential direction *ε*_*θθ*_. The incremental normal strain in the longitudinal direction Δ*ε*_*zz*_ was significantly higher than strain 0 except at a pressure range of 120–160 mmHg (Fig. 3b, d). There is no clear difference in this strain *ε*_*zz*_ between different radial positions (Fig. S5.1b).

The cumulative normal strain in the radial direction *ε*_*rr*_ was almost 0 except at a pressure range of 15–40 mmHg (Fig. 3a, c). Its incremental strain Δ*ε*_*rr*_ was not significantly different from strain 0 except at a pressure range of 15–40 mmHg (Fig. 3b, d). The strain Δ*ε*_*rr*_ had a positive value at a pressure range of 15–40 mmHg and was negative in other ranges (Fig. 3b, d). This result indicates that the aorta inflates in a low-pressure range and then becomes compressed in a high-pressure range. No clear difference was noticed in *ε*_*rr*_ between different radial positions (Fig. S5.1c).

**Fig. 4:**
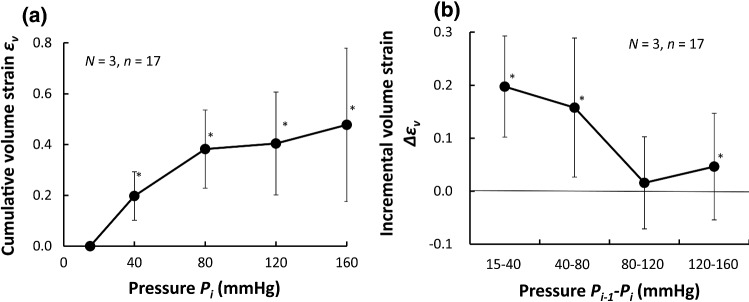
Volume strain during pressurization. **a** Cumulative and **b** incremental volume strain *ε*_*v*_. Incremental strain is expressed with Δ. *, *P* < 0.05 versus 0 strain. Data are shown as mean ± SD

The cumulative volume strain *ε*_*V*_ was about 38.3 ± 15.4% at 80 mmHg, and the incremental volume strain Δ*ε*_*V*_ at a pressure range of 80–120 mmHg was 1.6 ± 8.7% (Fig. 4).

### Shear strains

Figure S6.1 shows cumulative and incremental shear strains during pressurization. The results demonstrated that average shear strains were almost 0 for all pressure ranges, meaning that both positive and negative shear strains were equally present. Shear strains *ε*_*rθ*_ and *ε*_*rz*_ were not significantly different from strain 0.

**Fig. 5:**
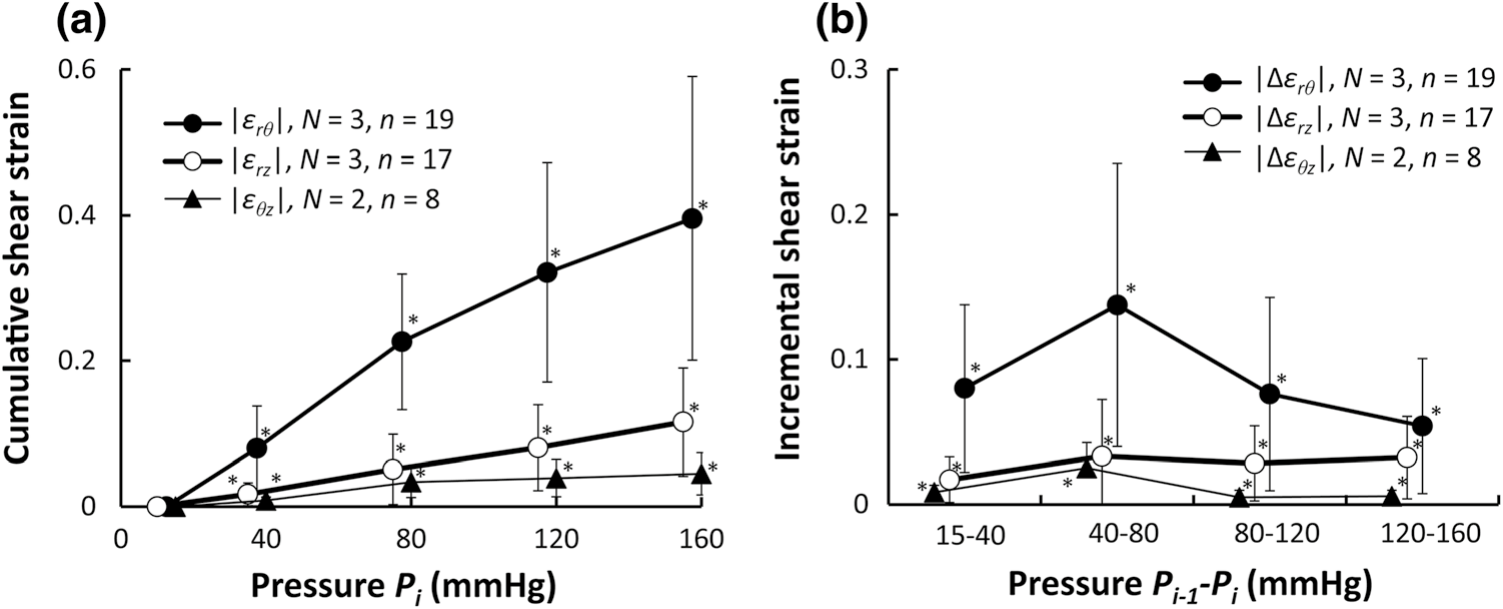
Changes in the absolute value of shear strains during pressurization. Cumulative and incremental shear strains are presented in (**a**, **b**), respectively. *ε*_*rθ*_: radial–circumferential shear strain, *ε*_*rz*_: radial–longitudinal shear strain*, ε*_*θz*_: circumferential–longitudinal shear strain. Incremental strains are expressed with Δ. *, *P* < 0.05 versus strain 0. Data are shown as Mean ± SD

Figure 5 plots the shear strains evaluated in the absolute values. An increase in the pressure resulted in a remarkable increase in the radial–circumferential shear strain |*ε*_*rθ*_|, a slight increase in the circumferential–longitudinal shear strain |*ε*_*rz*_|, and a negligible impact on the circumferential–longitudinal shear strain |*ε*_*θz*_| (Fig. 5a). All shear strains were significantly different from zero. For a pressure range of 80–120 mmHg, the incremental radial–circumferential shear strain |Δ*ε*_*rθ*_| was 7.6 ± 6.7% and the incremental circumferential–longitudinal shear strain |Δ*ε*_*θz*_| was 2.8 ± 2.6% (Fig. 5b). The absolute values of the shear strains |*ε*_*rθ*_|, |*ε*_*rz*_|, and |*ε*_*θz*_| showed no clear differences between radial positions (Fig. S5.1d–S5.1f).

### Principal strains

**Fig. 6:**
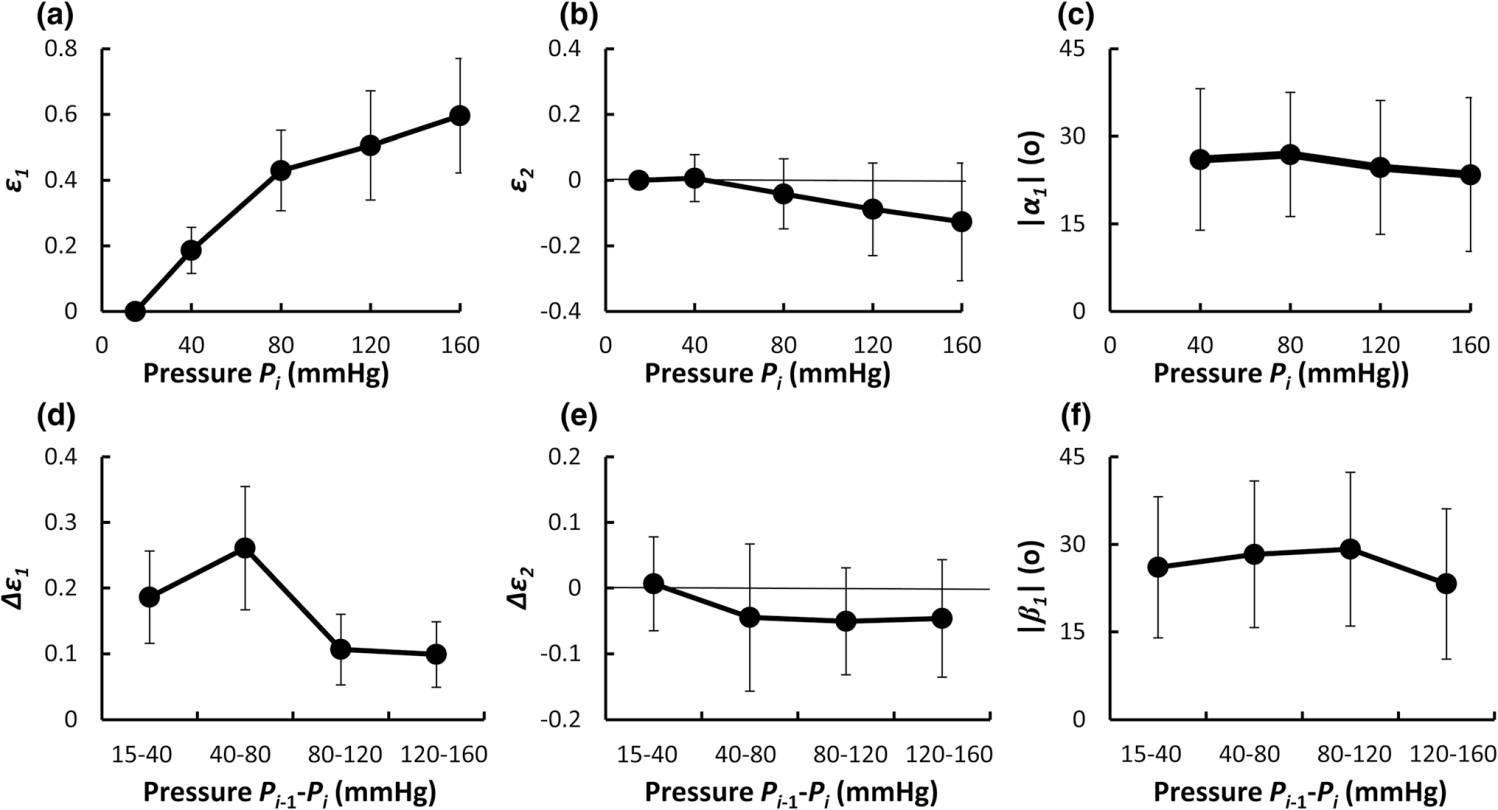
Changes in the principal strains in the radial–circumferential plane during pressurization. Upper row: **a** cumulative first principal strain *ε*_1_, **b** cumulative second principal strain *ε*_2_ and **c** angle of first principal direction |*α*_1_|. Lower row: **d** incremental first principal strain Δ*ε*_1_, **e** incremental second principal strain Δ*ε*_2_, and **f** angle of the first principal direction *β*_1_ at *P*_*i*_ defined in reference to configuration at *P*_*i*−1_. *N*umber of mice = 3; number of measured data = 19. Data are shown as mean ± SD

In the normal strain (Fig. 3) and shear strains (Fig. 5), the large strains were observed in the radial–circumferential plane. Thus, the principal strains were evaluated in the radial–circumferential plane as shown in Fig. 6. The first principal strain *ε*_1_ was larger with pressurization (Fig. 6a). In the physiological pressure range (from 80 to 120 mmHg), an incremental of the first principal strain was Δ*ε*_1_ = 10.6 ± 5.4% (Fig. 6d). The second principal strain *ε*_2_ slightly decreased with pressurization (Fig. 6b). In the physiological pressure range, an incremental of the second principal strain was Δ*ε*_2_ = − 5.1 ± 8.2% (Fig. 6e). Since both positive and negative shear strains were equally present in different locations at the same pressure load, the first principal direction was evaluated as their absolute value from the circumferential direction. The angle of the first principal direction over the whole pressure range showed |*α*_1_| = 23°–27° (Fig. 6c). The angle of the first principal direction from 80 to 120 mmHg was |*β*_1_| = 29° ± 13° (Fig. 6f).

### Strains in SMLs and ELs

**Fig. 7:**
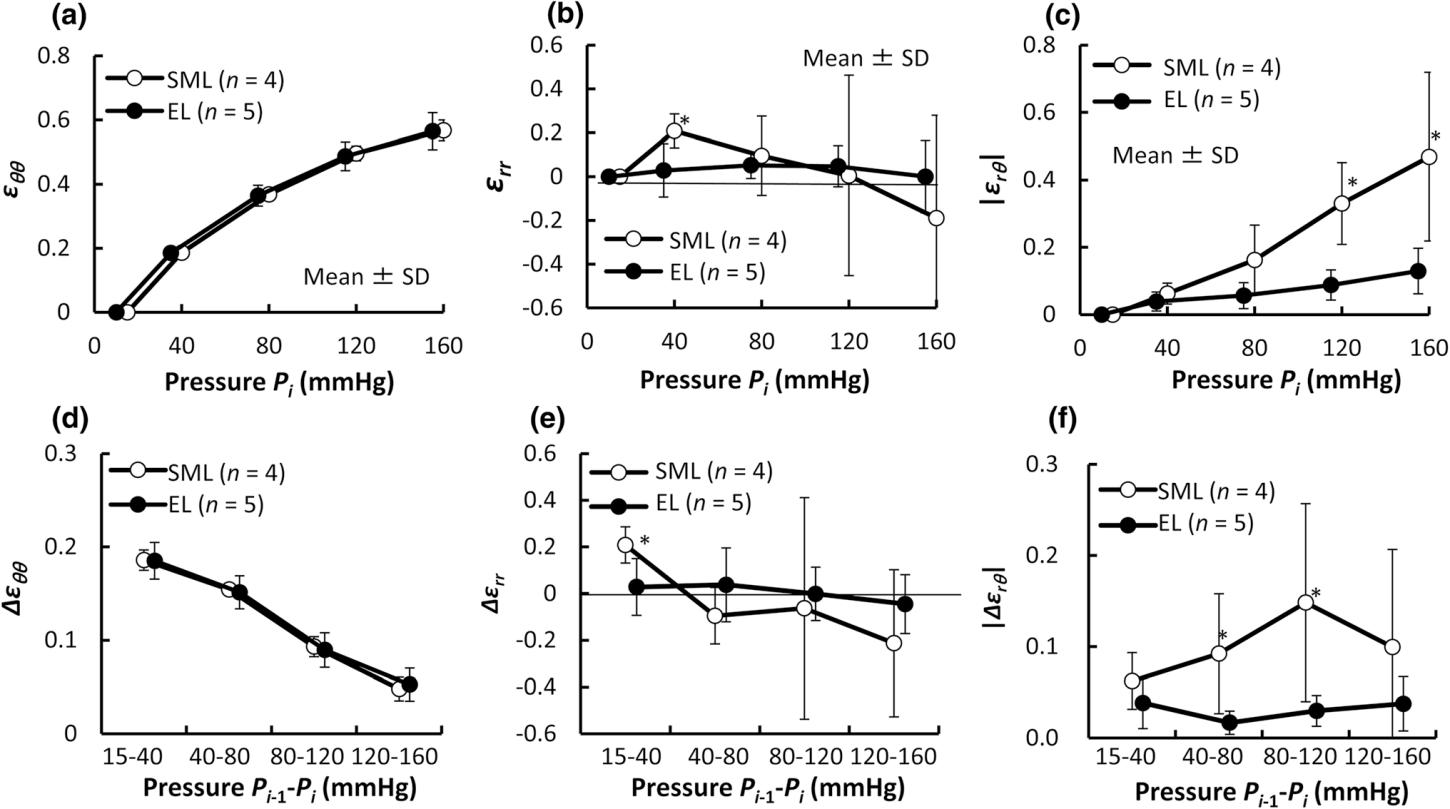
Changes in strains of SMLs and ELs in the radial–circumferential plane during pressurization. Upper row: **a** cumulative circumferential normal strain *ε*_*θθ*_, **b** cumulative radial normal strains *ε*_*rr*_ and **c** absolute value of radial–circumferential shear strain |*ε*_*rθ*_|. Lower row: **d** incremental circumferential normal strain Δ*ε*_*θθ*_, **e** incremental radial normal strain Δ*ε*_*rr*_, and **f** absolute value of incremental radial–circumferential shear strain |Δ*ε*_*rθ*_|. Data are shown as mean ± SD. *N*umber of mice = 1;*, *P* < 0.05 versus EL

Figure 7 shows strains of ELs and SMLs in the radial–circumferential plane. The circumferential normal strain *ε*_*θθ*_ increased nonlinearly with an increase in pressure. There was no significant difference in *ε*_*θθ*_ between SMLs and ELs (Fig. 7a, d). In contrast, the radial normal strain *ε*_*rr*_ of ELs showed a different trend from that of SMLs. The radial normal strain *ε*_*rr*_ in ELs remained almost 0 (Fig. 7b, e). On the other hand, *ε*_*rr*_ in SML increased until 40 mmHg, started to decrease, and became negative at 160 mmHg. This indicates that SMLs are gradually compressed in the radial direction with pressurization.

The absolute value of the radial–circumferential shear strain |*ε*_*rθ*_| increased with pressurization in both SMLs and ELs (Fig. 7c). The strain |*ε*_*rθ*_| in SMLs was significantly larger than that in ELs at 120 mmHg and above. A significant difference was also found in its incremental |Δ*ε*_*rθ*_| between SMLs and ELs for the ranges of 40–80 mmHg and 80–120 mmHg (Fig. 7f). Since the incremental strains |Δ*ε*_*rθ*_| in SMLs (14.8 ± 10.8%) were much larger in ELs (2.9 ± 1.6%) in the physiological pressure range, a radial–circumferential shear deformation was found to occur mainly in SMLs.

## Discussion

In our previous study, we found that collagen fibers in SMLs undulated less, and inferred that the aorta underwent a circumferential–radial shear strain (Sugita and Matsumoto 2017). According to the present study, the radial–circumferential shear strain |Δ*ε*_*rθ*_| of the aorta is 7.6 ± 6.1% (Fig. 5b) and the strain |Δ*ε*_*rθ*_| in SMLs (14.8 ± 10.8%) is larger than that in ELs (2.9 ± 1.6%) under physiological pressure (Fig. 7f). These data provide proof of our previous inference (Sugita and Matsumoto 2017). To our best knowledge, this is the first time a radial–circumferential shear deformation in the aorta is directly observed in the present study. The presence of the shear deformation implies that the aorta in vivo exposes SMCs in SML to radial–circumferential shear stresses. Interestingly, the average incremental of the radial–circumferential shear strain Δ*ε*_*rθ*_ was almost 0 (Fig. S6.1b). If the average shear strain represents the shear strain at a tissue level, the radial–circumferential shear strain is considered to be negligibly small at the tissue level although it could not be ignored at the cellular level. Since shear stress upregulates proliferation and differentiation of vascular SMC (Asada et al. 2005; Sterpetti et al. 1992), induced by pressure changes, shear stress on SMCs would play an important role of maintaining homeostasis of the aorta.

**Fig. 8:**
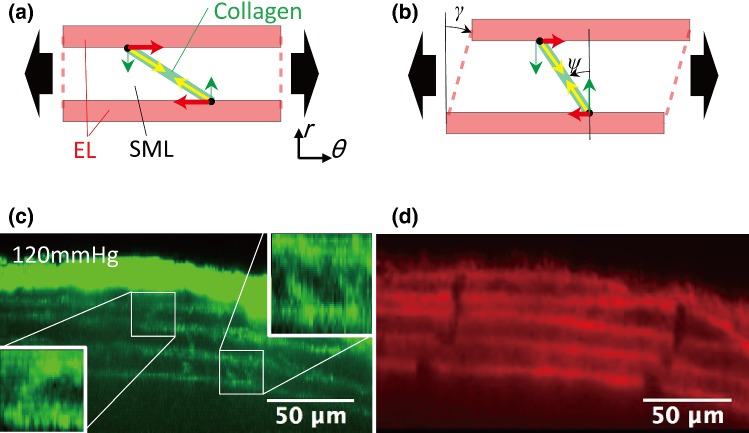
A possible mechanism of the radial–circumferential shear occurrence. **a** When a tensile force (black arrows) is exerted circumferentially to the aorta, collagen fibers bridging ELs are stretched and generate an elastic recoiling force (yellow arrows). A circumferential component of the elastic recoiling force (red arrows) gives radial–circumferential shear to SML. An example is given in (**b**) where a collagen fiber inclined counterclockwise *ψ* against the radial direction yields positive (clockwise direction) shear *γ*. Typical images of **c** collagen and **d** elastin fibers in the radial–circumferential plane at 80 mmHg. The elastin fiber image **d** demonstrates a positive shear in the first and second SMLs from the intimal side (bottom side) where collagen fibers are inclined counterclockwise (**c**). Negative shear was found in the third SML where collagen fibers lay clockwise

As shown in Fig. 5, radial–circumferential shear strains are present at the cell scale. Figure 8 schematically presents a possible explanation of why shear strains are generated in the aorta. When an intraluminal pressure is applied to an incompressible tube, its wall is stretched in the circumferential direction. This circumferential stretch force applies to a collagen fiber that obliquely bridges two adjacent ELs lying in the circumferential direction (Fig. 8a). Previously, we found that collagen fibers obliquely bridge tended to become undulated in low-pressure range of intraluminal pressure (Sugita and Matsumoto 2017). Thus, these collagen fibers become straight and pull the ELs in the fiber direction, and a circumferential component of the pulling force causes shear strain (Fig. 8b). According to this mechanism, the collagen fiber of a negative inclination angle (counterclockwise direction) against the radial direction (*ψ* in Fig. 8b) results in a positive (clockwise direction) shear strain (*γ* in Fig. 8b). In support of this model, images of the collagen fiber (Fig. 8c) and ELs (Fig. 8d) demonstrate that fibers of a positive angle result in negative radial–circumferential shear strain *ε*_*rθ*_ and vice versa.

The principal directions of strain are radial, circumferential, and longitudinal when the aorta is assumed to be a homogeneous cylindrical pipe with a linear elastic material, and intraluminal pressure is applied. At the tissue level, the principal directions are radial, circumferential, and longitudinal since the absolute values of the radial–circumferential, radial–longitudinal, and circumferential–longitudinal shear strains were almost 0 (Fig. S6.1). On the other hand, at a cellular level, the absolute value of the radial–circumferential shear strain |*ε*_*rθ*_| was different from 0 (Fig. 5a, d), which means that the principal direction at the cellular level was neither radial nor circumferential. Thus, SMCs in the aorta are not stretched only in the circumferential direction. Since the longitudinal normal strains *ε*_*zz*_ and Δ*ε*_*zz*_ were almost 0 (Fig. 3), the deformation of SMCs was confined mainly to the radial–circumferential plane. In the physiological pressure range, the angle of the first principal direction from 80 to 120 mmHg was |*β*_1_| = 29 ± 13° and the incremental principal strain Δ*ε*_1_ (ca. 10%, Fig. 6d) was twofold of the incremental circumferential normal strain Δ*ε*_*θθ*_ (ca. 5%, Fig. 3b). The tensile strain in vitro, considered to be physiological, applied to SMCs is 5% (Asanuma et al. 2003) to 10% (Balachandran et al. 2009) and was determined from changes in the aorta perimeter during a cardiac cycle (Thubrikar et al. 1980). The strain derived from the perimeter change corresponds to Δ*ε*_*θθ*_. Since Δ*ε*_1_ was twice as large as Δ*ε*_*θθ*_ according to the present study, more tensile strain should be applied to SMCs in studies in vitro to reproduce the mechanical environment of cells in the aorta.

SMCs in the aorta extend obliquely (Dingemans et al. 2000; Fujiwara and Uehara 1992). According to Fujiwara and Uehara (Fujiwara and Uehara 1992), the average angle of extension is 23° for the rat thoracic aorta. This value is close to the angle of the first principal direction in the radial–circumferential plane |*β*_1_| (29° ± 13° from the circumferential direction) shown in Fig. 6c. Reportedly, cultured in a 3D gel and cyclically stretched, SMCs align in the stretch direction (Kanda et al. 1993). These results suggest that SMCs in the aorta extend in the direction of the largest strain.

Mainly composed of water, the aortic tissue is often assumed to be incompressible (Giannakoulas et al. 2005; Singh et al. 2015; Zidi and Allaire 2015). However, the present results do not support this assumption. During pressurization, the volume strain at 80 mmHg is *ε*_*V*_ = 38.3 ± 15.4%, indicating an increase in the volume of the aortic tissue. Because the volume strain is a product of *ε*_*zz*_, *ε*_*θθ*_, and *ε*_*rr*_, overestimation of strains would increase the volume strain. As discussed in a later paragraph, measurement error can be the most likely to occur in *ε*_*rr*_. To check measurement accuracy in the radial direction, we measured a distance between the luminal surface and the outer surface of the aorta as the whole thickness and evaluated a radial normal strain from 15 mmHg to 80 mmHg. The results showed a radial normal strain of +3.9 ± 11.4% (*N* = 4) representing slight thickening or almost no change, although one specimen showed thinning (− 10.7%). This radial normal strain is similar to the one obtained with the method described in Supplementary Material S3 (2.5% ± 13.3%). Consequently, we got the volume strain *ε*_*V*_ of 36.8% ± 10.4%. This is quantitatively in a good agreement with *ε*_*V*_ of 38.3 ± 15.4% described in 3.2. These results demonstrate that measurement errors in the calculated strains are not large enough in estimating the volume strain. Moreover, when we implemented a uniaxial stretch of a silicone rubber sheet using the similar experimental setup, we found a decrease in the thickness of the sheet with stretch (see Supplementary Material S7). Although the Poisson’s ratio calculated for the sheet was 0.55 due probably to errors in determining the initial thickness at zero load state, the uniaxial stretch corroborated the validity and accuracy of the current experiment setup in measurement of the thickness. It would be therefore factual that the thickness of the aorta did not decrease during pressurization. The expansion of the aortas was also reported by Nolan et al. (Nolan and McGarry 2016). They stretched aortic specimens in the radial direction by 28% and found about 9.31% increase in the volume. Although their value cannot be compared with our data due to the difference in the methodology, the expansion of the aorta was confirmed in other study, too. The present study extends the findings of Nolan et al. (2016) such that the aortic expansion occurs even in the physiological state. It is speculated that such an increase in the volume is attributed to liquid transport from the lumen of the aorta to the aortic tissue driven by a transmural pressure gradient. In hypertension, the pressure gradient becomes steeper and more transport occurs. Enrichment of nutrients and elevation of flow shear in tissues would change cell metabolism, leading to excessive growth and dysfunction.

The aortic tissue might have been partially damaged in the process of photo-bleaching. Jayyosi et al. (2014) reported that this technique was nondestructive and did not have any impact on mechanical properties. In their study, an SHG signal in a potential burnt area was detectable. This suggested that the collagen fibers did not suffer from photo-bleaching and appeared intact. Looking at our samples, we found a loss of SHG signals in the photo-bleached area, implying that collagen fibers were damaged to some extent. To investigate what impact the damage of collagen fibers has on a strain field, we performed an additional experiment. A tissue sample was newly prepared, and strain markers were created by photo-bleaching with utmost care so that collagen fibers were minimally damaged. After the strains were measured using the same method described in “Strain analysis” section, a laser was applied to the photo-bleached area to burn the collagen fibers, and the strains were measured again (see Supplementary Materials S8). A comparison of these strains demonstrated almost the same normal strain, indicating that the damage of collagen fibers had little impact on the measured strain field.

The standard deviations of the radial normal strain *ε*_*rr*_ were larger than those of the circumferential *ε*_*θθ*_ and longitudinal strains *ε*_*zz*_ (Fig. 3). This is probably due to measurement accuracy of a distance between markers. Length between two neighboring markers in the radial direction was approximately 20 pixels, and a measurement error of 1 pixel results in a change of the radial strain *ε*_*rr*_ of 0.05. Length between two neighboring markers in the circumferential and longitudinal directions was approximately 250 pixels, and a measurement error of 1 pixel results in a change of strain of 0.004 (*ε*_*θθ*_ and *ε*_*zz*_). Thus, *ε*_*rr*_ is more sensitive to measurement errors than the circumferential strain *ε*_*θθ*_ and the longitudinal strain *ε*_*zz*_. Even taking account of these changes in the strains, changing trends of the magnitude of the strains against the pressure shown in Fig. 3 would not vary. Changes in the shear strain *ε*_*rθ*_ due to the 1 pixel movement were typically 0.007 and 0.024 at maximum. Similarly, changes in the shear strain *ε*_*rz*_ due to the 1 pixel movement were typically 0.003 and 0.012 at maximum. These errors therefore would have negligible impacts on the results shown in Figs. 5 and 7c. These error analyses indicate that our conclusion remains the same although some errors occurred in the measurement.

Shear deformations in the aortic wall during pressurization may be concerned with aortic dissection. It most often begins with the intimal tear in the aortic intima and media. Blood surges through the tear, causing distal propagation of separation of the layers along the length of the vessel. Although the etiology of the thoracic aortic dissection still remains unclear, it has been thought that the dissection results from a combination of hemodynamic stresses, degeneration of the aortic media including loss of smooth muscle cells and fragmentation and depletion of elastic fibers (He et al. 2006) in addition to genetic predisposition and epidemiological risk factors (Wu et al. 2013). Delamination tests of thoracic aortic aneurysm revealed that aneurysms had lower strength than non-aneurysmal aortas (Pasta et al. 2012, 2016). If shear deformation occurs at vulnerable sites in the aortic wall and exceeds allowable shear strain, ELs tears from SMLs and the aortic dissection begin from these sites. Lu et al. (2003) and Garcia et al. (2013) externally and forcedly applied a twisting force to arteries around the longitudinal axis to provide mechanical properties of blood vessels under torsion. Because a type of applied forces and shear strains described in their studies (circumferential–longitudinal shear strain) are different from the ones in the present study (radial–circumferential shear strain), no further discussions on the contribution of the shear deformation to the initiation of the aortic dissection can be made. However, their methods are helpful to evaluate allowable shear strain of the aortic media under blood pressure in discussing the implications of the radial–circumferential shear strain in the initiation of the aortic dissection. While clinical applications of knowledge obtained in the present analysis is still challenging, the present analysis will help explore the mechanism of the initiation of aortic aneurysms.

There are several limitations in this study to consider the aorta in an in vivo state. First, quasi-static pressurization is different from the in vivo state where aortas undergo dynamic deformations due to heart beat. In the dynamic deformations, strains are expected to be smaller than the present results because incremental in the elastic modulus of thoracic aorta in the dynamic deformation is larger than that in the static deformation (Learoyd and Taylor 1966). Second, cell activities were not considered. All the experiments were conducted at a room temperature. Although cells in specimens were kept in a KH solution, it is possible that activity of the cells was different from those in an in vivo state. Reportedly the stiffness of arteries is concerned with the active state of smooth muscle (Handa 1983). Reduced cell activity would have resulted in softening of the aorta and so unphysiological strains in the aortic wall. Third, the present study analyzed only the ventral side of the aorta. As intercostal arteries and back born are present behind the aorta, the dorsal side has different environments from the ventral side. The intercostal arteries and back born may constrain the movement of the aorta during pressurization and give rise to different mechanical properties in the dorsal side.

## Conclusion

We observed the 3D strain of aortic tissue during pressurization at the cell scale. Several important results obtained in this study are: (1) SMLs, at the cell scale, experience radial–circumferential shear strain during pressurization, including under physiological pressure; (2) averaged radial–circumferential, radial–longitudinal, and circumferential–longitudinal shear strains are almost 0, indicating that the principal strain direction is radial, circumferential, and longitudinal at the whole aortic wall level; (3) the first principal strain direction in the radial–circumferential plane was 29 ± 13° from the circumferential direction; (4) the aortic walls are not an incompressible material. As cellular strain could serve as tensional homeostasis of cells, detailed and comprehensive inspection of the mechanical environment of the aorta has significant implications to understand aortic health and disease.

## Electronic supplementary material

Below is the link to the electronic supplementary material.

Supplementary material 1 (DOCX 3533 kb)

**Movie 1** Deformation of ELs during pressurization. Image contrast was adjusted for clear visibility. (AVI 178 kb)
