# Supplementary material for: Three-dimensional analysis of the thoracic aorta microscopic deformation during intraluminal pressurization
Source: Biomech Model Mechanobiol. 2019 Jul 11;19(1):147–57. doi: 10.1007/s10237-019-01201-w (PMC7005079; doi:10.1007/s10237-019-01201-w)
Supplement: Supplementary file 1 — Supplementary material 1 (DOCX 3533 kb) [file 10237_2019_1201_MOESM1_ESM.docx]

**Supplementary material**

**Title:**

**Three-dimensional analysis of the thoracic aorta microscopic deformation during intraluminal pressurization**

Shukei Sugita^1^, Masaya Kato^1^, Fukui Wataru^1^, and Masanori Nakamura^1^

**Affiliation:**

1 Biomechanics Laboratory, Department of Electrical and Mechanical Engineering, Graduate School of Engineering, Nagoya Institute of Technology, Gokiso-cho, Showa-ku, Nagoya 466-8555, JAPAN

**S1. Preprocessing of images**

To analyze 3D strains, captured images were pre-processed as shown in Fig. S1.1.

**
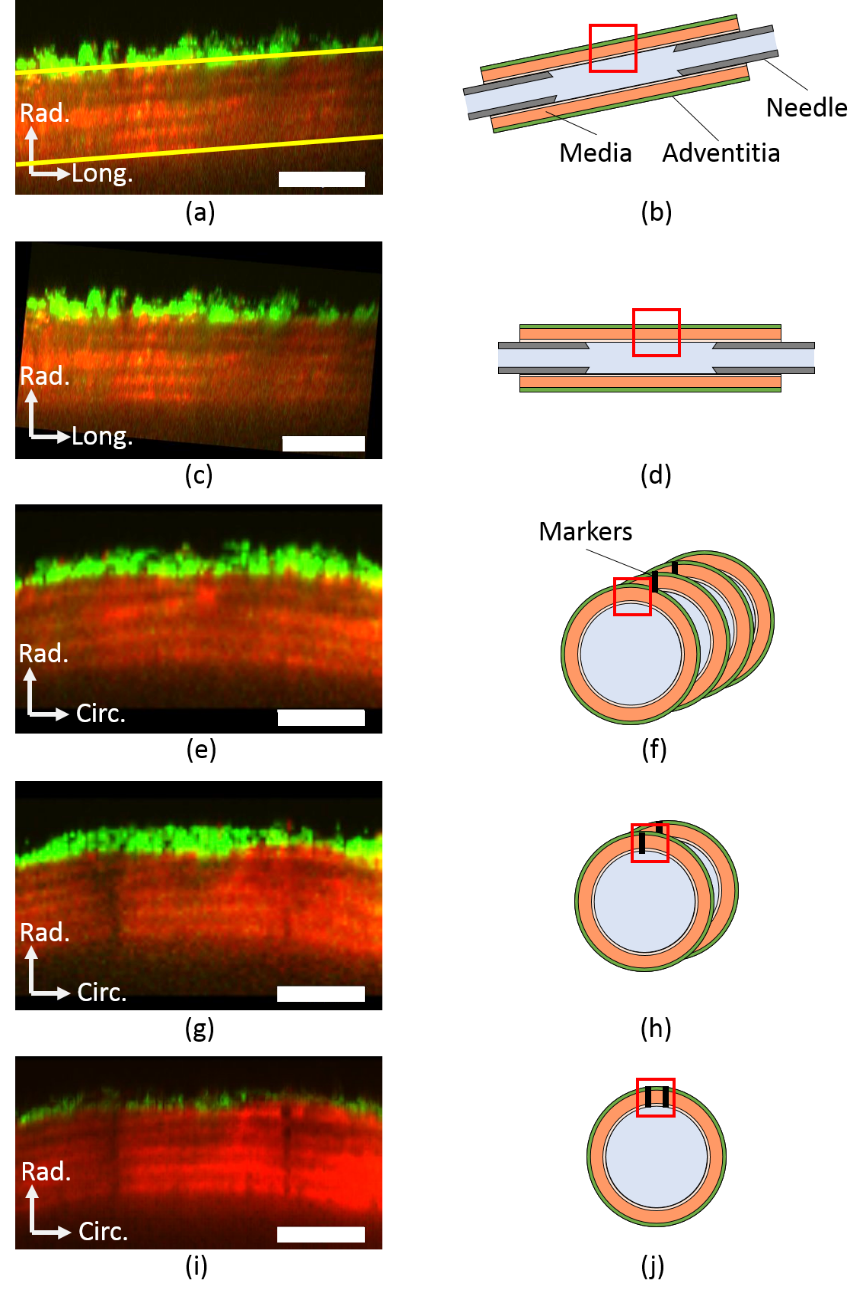
**

**Fig. S1.1** Image pre-processing for strain analysis. Captured images (a, c, e, g, and i) and corresponding illustrations (b, d, f, h, and j) are shown on the left and right side, respectively. A red box in the illustration represents the location of the captured image on its left. The radial-longitudinal image stack (a and b) was rotated to align the longitudinal direction of the aorta (yellow lines) to the horizontal direction (c and d). After reslicing into the radial-circumferential images (e and f), images showing the markers were selected (g and h), and their minimum intensity projection image was obtained (i and j). Image contrast was adjusted for visibility. Bars in images = 50 µm.

**S2. Determining node positions in bleached markers**

Nodes to evaluate tissue deformation (Figs. 3–5) were determined in bleached markers through the following steps illustrated in Fig. S2.1. A straight line passing through the center of EL was firstly drawn in the circumferential direction in a radial circumferential plane (Fig. S2.1a), and intensity was profiled along the line (Fig. S2.1b). A characteristic point in the intensity profile was manually selected. In this image (Fig. S2.1b), a point with the minimum intensity was chosen (red arrow in Fig. S2.1b).

To evaluate strains in ELs and SMLs separately (Fig. 7), one of four mice was used for the analysis of local strain of ELs and SMLs. Lines were drawn circumferentially in bleached markers at the most adventitial (solid line) and intimal (broken line) edges of each EL (Fig. S2.1c), and the intensity profiles were obtained along the lines. Then, a characteristic point was selected in the intensity profile as a node to be used for assessing strains of ELs and SMLs.


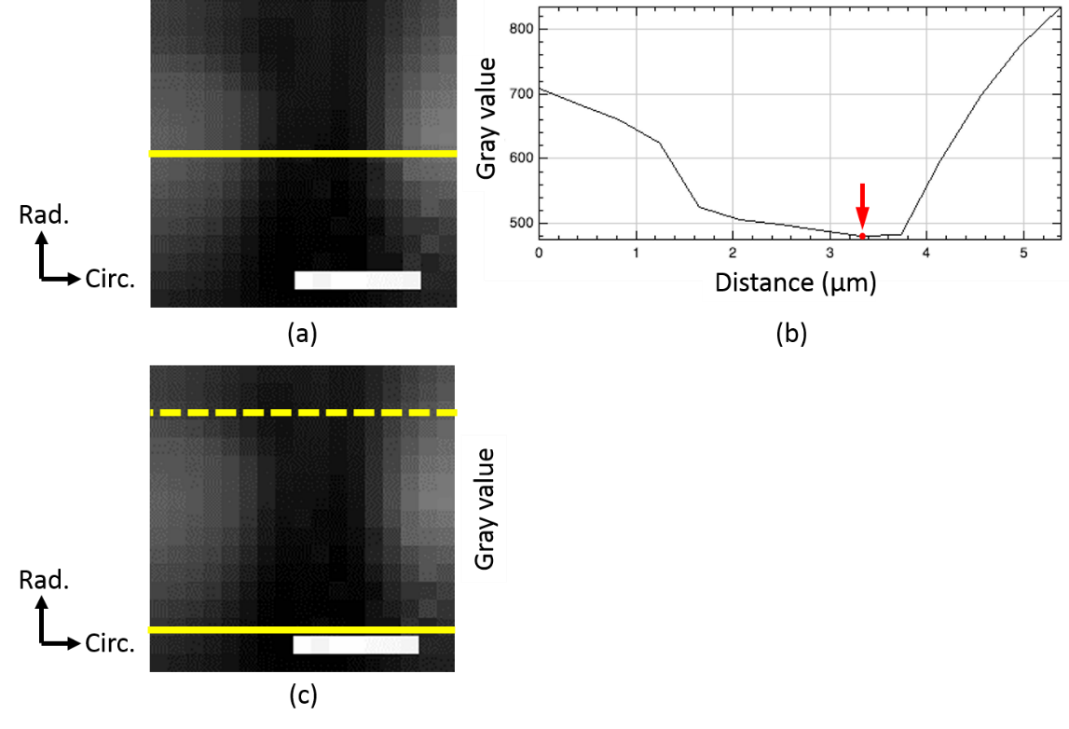


**Fig. S2.1** Method for determining node positions in photo-bleached markers. (a and b) From a marker image (a), gray value profiles on the analysis line (yellow line in (a)) were obtained as shown in (b). Red point (arrowed) in (b) shows the selected point. (c) To evaluate strains in ELs and SMLs, lines were laid in the circumferential direction at the most adventitial (solid line) and intimal (broken line) edges of each EL. Bars in the panel (a) and (c) correspond to 5 µm.

**S3. Strain calculation**

Strains were calculated in radial-circumferential, radial-longitudinal, and circumferential-longitudinal planes (Fig. S3.1a) using isoparametric mapping. In each plane, four nodes *i*, *j*, *k*, and *l* were selected according to the method described in S2. Displacements (*u_n_*, *v_n_*) of nodes *n* = *i*, *j*, *k*, and *l* between consecutive pressures were measured (Fig. S3.1b, S3.1c). A quadrangle element made by the four nodes was mapped to the square which has nodes at (± 1, ± 1) in the *ξ*-*η* plane (Fig. S3.1d). Quantities including coordinates (*x*, *y*) and its displacement (*u*, *v*) consecutive pressures in the quadrangle element are written as:

 (S3.1)

where *φ* = *x*, *y*, *u*, and *v* and *s*hape functions *N_i_*, *N_j_*, *N_k_* and *N_l_* are given by

$\left.\begin{matrix} N_{i}=\frac{1}{4}\left(1-\xi\right)\left(1-\eta\right) & N_{j}=\frac{1}{4}\left(1+\xi\right)\left(1-\eta\right) \\ N_{k}=\frac{1}{4}\left(1+\xi\right)\left(1+\eta\right) & N_{l}=\frac{1}{4}\left(1-\xi\right)\left(1+\eta\right) \end{matrix}\right\}$ (S3.2)

Incremental normal strains *Δε_xx_* in the *x*-direction and *Δε_yy_* in the *y*-direction and incremental shear strain in the *y*-direction at the surface perpendicular to the *x*-direction *Δε_xy_* in the *x-y* plane are defined as

$\left\{\begin{matrix} \Delta\varepsilon_{xx} \\ \Delta\varepsilon_{yy} \\ \Delta\varepsilon_{xy} \end{matrix}\right\}=\left\{\begin{matrix} \frac{\partial u}{\partial x} \\ \frac{\partial v}{\partial y} \\ \frac{1}{2}\left(\frac{\partial u}{\partial y}+\frac{\partial v}{\partial x}\right) \end{matrix}\right\}$. (S3.3)

Strain components in (S3. 3) are obtained by

$\left[\begin{matrix} \frac{\partial\kappa}{\partial x} \\ \frac{\partial\kappa}{\partial y} \end{matrix}\right]=\left[\begin{matrix} \frac{\partial x}{\partial\xi} & \frac{\partial y}{\partial\xi} \\ \frac{\partial x}{\partial\eta} & \frac{\partial y}{\partial\eta} \end{matrix}\right]^{-1}\left[\begin{matrix} \frac{\partial\kappa}{\partial\xi} \\ \frac{\partial\kappa}{\partial\eta} \end{matrix}\right]$. (S3.4)

where *κ* = *u* and *v*. From Eqs. (S3.1) to (S3.4), strains (*Δε_θθ_*, *Δε_rr_*, *Δε_rθ_*), (*Δε_zz_*, *Δε_rz_*) and *Δε_θz_* were calculated in the radial-circumferential, radial-longitudinal and circumferential-longitudinal planes, respectively, for instance by replacing *x* and *y* with *r* and *θ* in the radial-circumferential plane. In evaluating tissue deformation, strains at (*ξ*, *η*) = (0, 0) were used as representatives.

Volume strain *Δε_v_* was calculated as

$\Delta\varepsilon_{V}=\left(1+\Delta\varepsilon_{\theta\theta}\right)\left(1+\Delta\varepsilon_{zz}\right)\left(1+\Delta\varepsilon_{rr}\right)-1$. (S3.5)

**
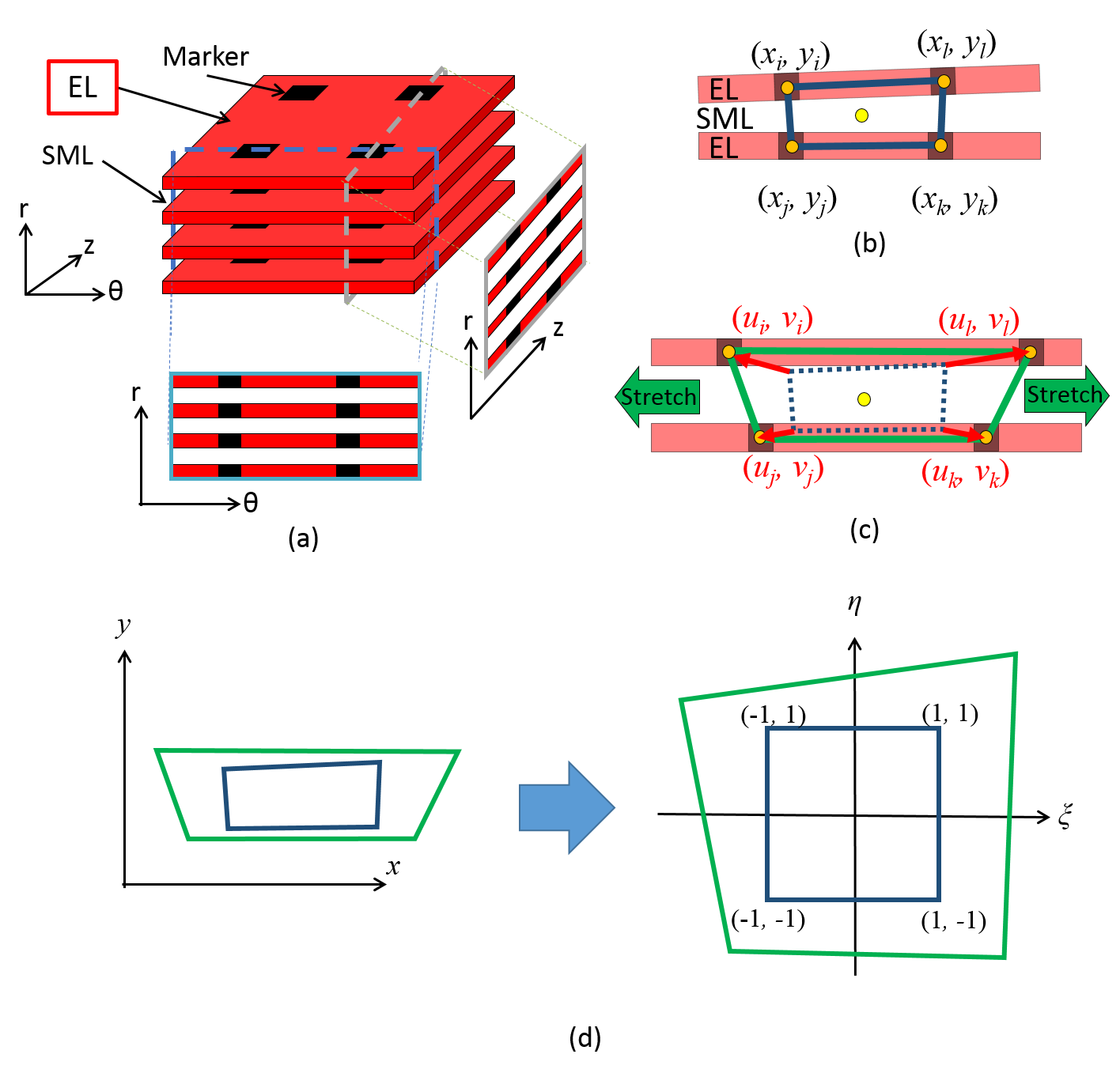
**

**Fig. S3.1** Strain analysis by isoparametric mapping. In each plane (a), coordinates of four markers (b) and their displacements (c) were measured. (d) Incremental strains *Δε_mn_*(*P_i_*_-1_, *P_i_*) with *m* and *n* = *θ*, *r*, and *z* were calculated from displacements between the consecutive pressures, *P_i_*_-1_ and *P_i_*. Configurations at *P_i-1_* and *P_i_* are shown in blue and green lines, respectively. Configurations in the *x*-*y* plane were mapped onto the *ξ*-*η* plane to calculate the incremental strain.

**S4. Derivation of the cumulative strain, Equation (1)**

Let consider a stretch ratio *λ_mn_*(*P_i_*) at pressure *P_i_* from the pressure level of *P_0_* = 15 mmHg. Here,

$\lambda_{mn}\left(P_{0}\right)=1$. (S4.1)

If an incremental of the stretch ratio of the aorta from *P_0_* to *P_1_* is *Δλ_mn_*(*P_0_*, *P_1_*), the stretch ratio at *P_1_* = 40 mmHg, *λ_mn_*(*P_1_*), is obtained by

$\lambda_{mn}\left(P_{1}\right)=\lambda_{mn}\left(P_{0}\right)\times\Delta\lambda_{mn}\left(P_{0}, P_{1}\right)$. (S4.2)

Similarly, when the stretch ratio from *P_i-1_* to *P_i_* pressure is given by *Δλ_mn_*(*P_i_*_-1_, *P_i_*), the stretch ratio at *P_i_* is

$\lambda_{mn}\left(P_{i}\right)=\lambda_{mn}\left(P_{i-1}\right)\times\Delta\lambda_{mn}\left(P_{i-1}, P_{i}\right)$. (S4.3)

Since the cumulative the stretch ratio *λ_mn_*(*P_i_*) is {1 + *ε_mn_*(*P_i_*)} and the incremental of the stretch ratio *Δλ_mn_*(*P_i_*_-1_, *P_i_*) is {1 + *Δε_mn_* (*P_i_*_-1_, *P_i_*)}, Eq. (S4.3) is equal to

$1+\varepsilon_{mn}\left(P_{i}\right)=\left\{1+\varepsilon_{mn}\left(P_{i-1}\right)\right\}\left\{1+\Delta\varepsilon_{mn}\left(P_{i-1}, P_{i}\right)\right\}$ (S4.4)

Rearrangement of Eq. (S4.4) yields Eq. (1).

**S5. Difference in strains between ELs within a single specimen**

ELs were numbered radially from the adventitial to intimal side, and average and SD of strains in each radial position were calculated. Figure S5.1 shows normal and shear strains at various radial positions. No clear tendency and differences were obtained when samples were compared in each radial position.


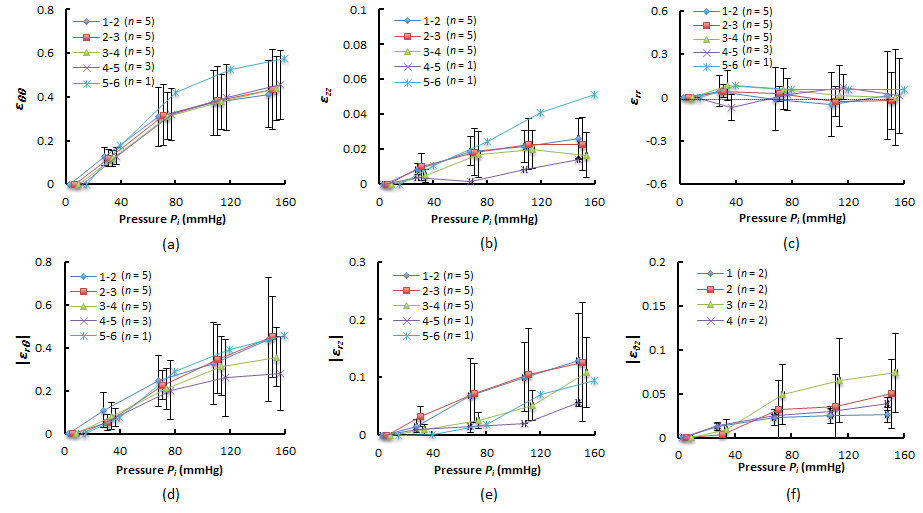


**Fig. S5.1** Cumulative strains at various radial positions. (a–c) Normal strains in the (a) circumferential *ε_θθ_*, (b) longitudinal *ε_zz_*, and (c) radial direction *ε_rr_*. (d–f) Absolute values of (d) the radial-circumferential shear strains |*ε_rθ_*|, (e) the radial-longitudinal shear strains |*ε_rz_*|, and (f) the circumferential-longitudinal shear strains |*ε_θz_*|. Data are shown as Mean ± SD. Hyphened-numbers in legends represent the radial positions of ELs numbered serially from the adventitial side.

**S6. Shear strains**

Figure S6.1 shows radial-circumferential shear strain *ε_rθ_*, radial-longitudinal shear strain *ε_rz_*, and circumferential-longitudinal shear strain *ε_θz_*. Overall, the average of shear strains was almost 0. As seen in Fig. 2b, some ELs moved to the right relative to adjacent ones at the intimal side, and others moved to the left, yielding both positive and negative shear strains, respectively. As a consequence, their average results in shear strain is close to 0.


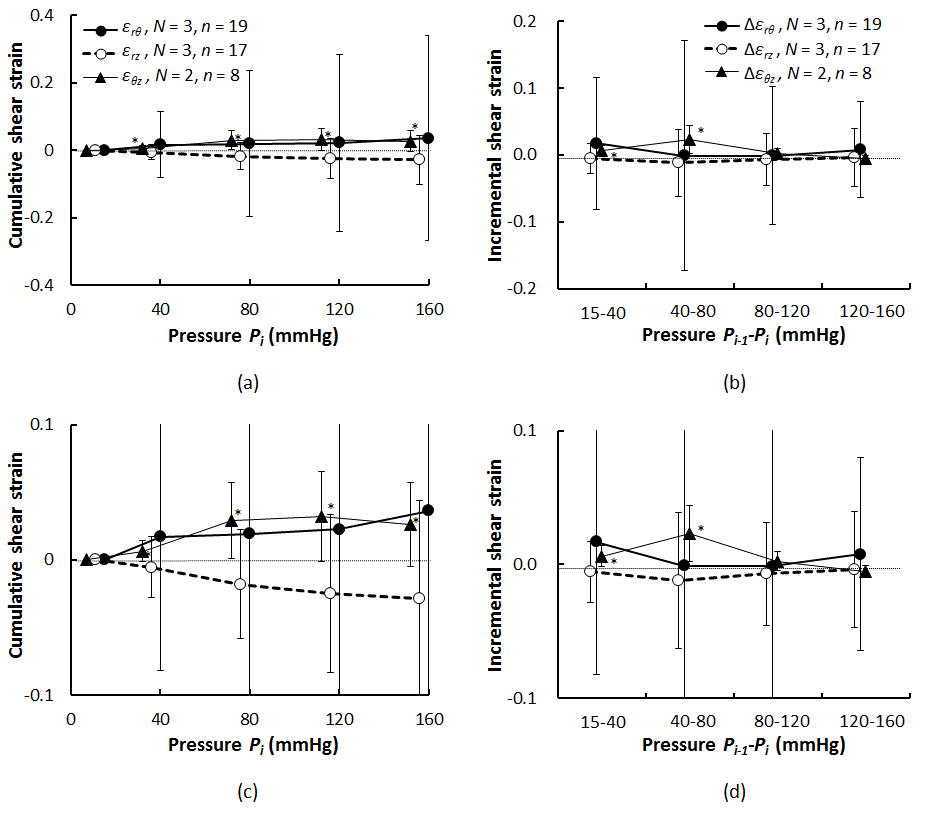


**Fig. S6.1** Shear strain during pressurization. (a) Cumulative and (b) incremental radial-circumferential shear strain *ε_rθ_*, radial-longitudinal shear strain *ε_rz_*, and circumferential-longitudinal shear strain *ε_θz_*. The panel (c) and (d) are the ones, in which vertical axis in the panel (a) and (b), respectively, was magnified. Incremental strain is expressed with Δ. *, *P* < 0.05 vs 0 strain. Data are shown as Mean ± SD.

**S7. Uniaxial tensile test of a silicone rubber sheet under a two-photon microscope**

To confirm that the thickness was measured with a certain accuracy, we carried out a uniaxial tensile test on a silicone rubber sheet whose Poisson’s ratio is known. Schematic illustration of observation method is shown in Fig S7.1a. On both sides of the sheet (*x* × *y* × *z* = 10 mm×96 mm×0.2 mm), 1 µg/µL CM-DiI (C7000, Invitrogen) in DMSO (043-07216, Wako) was applied as a maker. Then the sheet was stretched in the *y* direction. At every 5% strain, the fluorescent markers were imaged under the two photon microscope described in the “2.3 Two-photon and second harmonic generation light microscopy” section. In this imaging, 575–630 nm of bandpass filter (FV-10-MRG/R, Olympus) and laser power of 2.0% was used. Figure S7.1b–S7.1h shows the image of the fluorescent dye applied to the silicone sheet. As being stretched, a distance between the surfaces got smaller, showing a decrease in the thickness (*z*) of the sheet. Figure S7.1i plots a strain in the thickness direction against a strain of the uniaxial stretch. A Poisson’s ratio calculated as a slope of a linear regression line for Fig. S7.1i was 0.55. This is almost equal to the known Poisson’s ratio of silicone rubber (0.5), although it is larger than 0.5 due to measurement errors of the initial thickness (thickness at zero stretch). These results suggest that the present measurement method for the thickness of aortas have a certain accuracy.


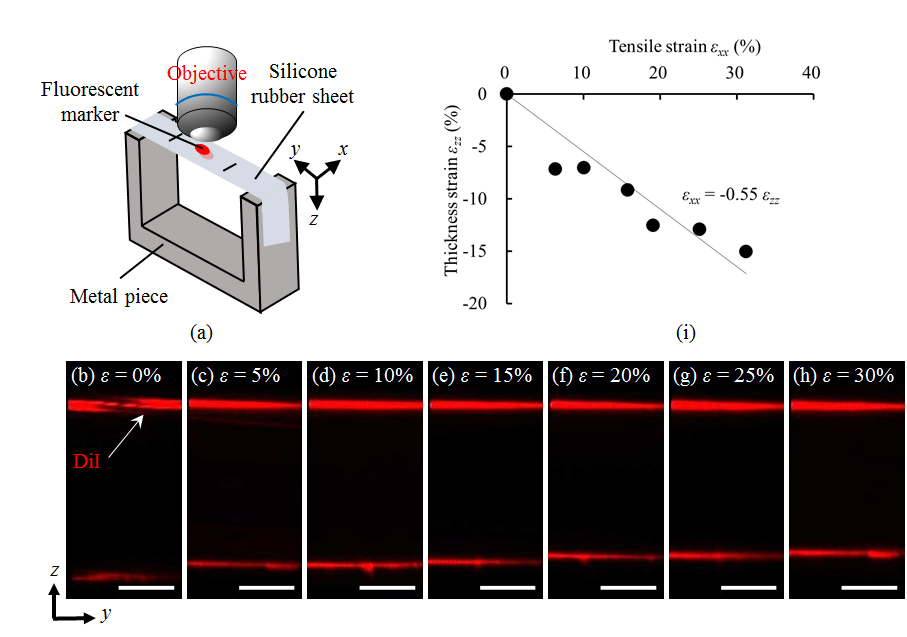


**Fig. S7.1** Confirmation of thickness changes in specimen under multiphoton microscope. (a) Schematic illustration of experimental setup. (b–h) Resliced images of fluorescent dye painted on the surface of silicone rubber sheet under a two-photon microscope at tensile strain of (b) 0, (c) 5, (d) 10, (e) 15, (f) 20, (g) 25, and (h) 30%. Bars = 30 µm. Silicone specimen stretched in the *y* direction was imaged from *z* direction. (i) Relationship between thickness of silicone rubber sheet-tensile strain.

**S8. Effect of markers made by holes on the strain**

Jayyosi et al. (Jayyosi et al. 2014) addressed the usefulness of creating photo-bleached markers to measure the aorta deformation. Although it was anticipated that elastin and collagen fibers suffered from photobleaching, their image analysis revealed that elastin fiber networks did not appear modified and SHG signals of collagen fibers in a photo-bleached area were detectable. These observations suggested that elastin and collagen fibers remained structurally preserved. The same procedure by Jayyoshi et al. (2014) was first followed in our experiments. However, it turned out that markers in resliced images appeared so unclear that we failed to track them during tissue deformations. We therefore decided to increase the laser power. Consequently, the markers became clearly visible while we observed a loss of SHG signals. To evaluate the influence of tissue damage caused by the laser on tissue deformation, we carried out additional experiments. First, we created markers with a weak laser power that kept SHG signals of collagen fibers intact. Although the markers were hard to recognize in this setting, we managed to spot them in two layers in one sample (Fig. S8.1a). As the SHG signals were still present, it implied that collagen fibers were mostly intact. We then measured the strains and once measured, a laser was additionally applied to the marker sites. As seen in Fig. S8.1b, SHG signals were no longer detected in the marker positions. Figure S8.1c and S8.1d show resliced images of the markers in the radial-circumferential plane before and after the additional laser application, respectively. Figure S8.1e and S8.1f compares circumferential and radial normal strains *Δε_θθ_* and *Δε_rr_* between before and after the additional laser application, respectively. As seen, the strains were similar regardless of the presence of SHG signals. These results indicate that tissue damages caused by the laser used in this study have little influence on tissue deformation.

**
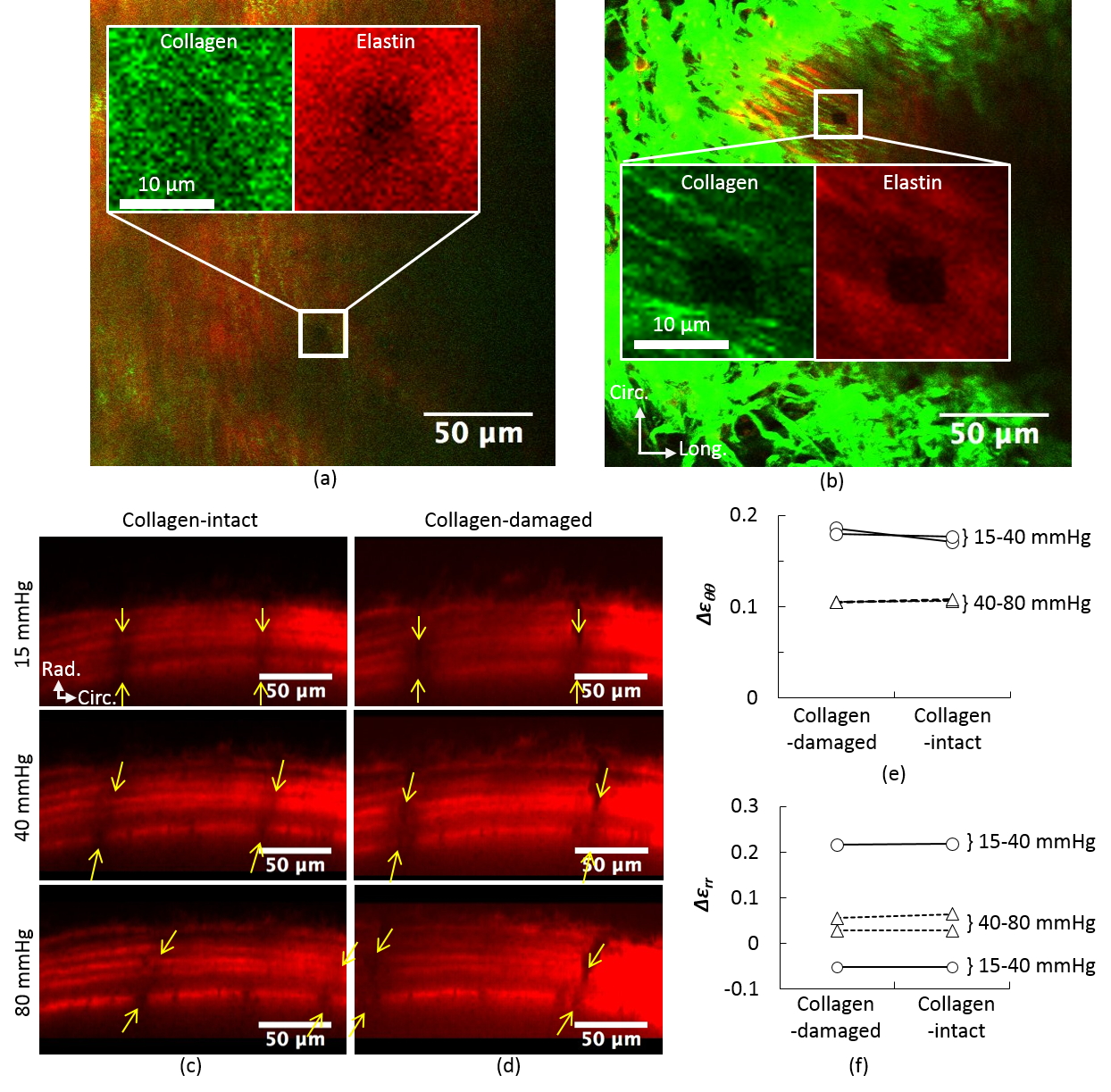
**

**Fig. S8.1** Comparison of normal strains measured using collagen-intact and collagen-damaged markers. (a and b) Typical elastin in two-photon (red) and collagen in SHG light (green) images (a) with collagen-intact and (b) collagen-damaged marker. Radial-circumferential plane images of ELs (c) with collagen-intact and (d) collagen-damaged marker. Yellow arrows indicate markers which were used for strain measurement. (e and f) Normal strain in the (e) circumferential and (f) the radial directions at 15–40 mmHg and 40–80 mmHg.

**References**

Jayyosi C, Fargier G, Coret M, Bruyère-Garnier K (2014) Photobleaching as a tool to measure the local strain field in fibrous membranes of connective tissues Acta Biomater 10:2591-2601 doi:https://doi.org/10.1016/j.actbio.2014.02.031
